# Association between the risk and severity of Parkinson’s disease and plasma homocysteine, vitamin B12 and folate levels: a systematic review and meta-analysis

**DOI:** 10.3389/fnagi.2023.1254824

**Published:** 2023-10-24

**Authors:** Yuxin Quan, Jisen Xu, Qing Xu, Zhiqing Guo, Ruwei Ou, Huifang Shang, Qianqian Wei

**Affiliations:** ^1^West China School of Medicine, West China Hospital, Sichuan University, Chengdu, Sichuan, China; ^2^State Key Laboratory of Biotherapy, Sichuan University, Chengdu, Sichuan, China; ^3^Department of Neurology, Laboratory of Neurodegenerative Disorders, West China Hospital, Sichuan University, Chengdu, Sichuan, China

**Keywords:** Parkinson’s disease, homocysteine, vitamin B12, folate, metaanalysis, systematic review

## Abstract

**Background:**

Parkinson’s disease (PD) is recognized as the second most prevalent progressive neurodegenerative disease among the elderly. However, the relationship between PD and plasma homocysteine (Hcy), vitamin B12, and folate has yielded inconsistent results in previous studies. Hence, in order to address this ambiguity, we conducted a meta-analysis to summarize the existing evidence.

**Methods:**

Suitable studies published prior to May 2023 were identified by searching PubMed, EMBASE, Medline, Ovid, and Web of Science. The methodological quality of eligible studies was assessed using the Newcastle-Ottawa Quality Assessment Scale (NOS). Meta-analysis and publication bias were then performed using R version 4.3.1.

**Results:**

The results of our meta-analysis, consisting of case–control and cross-sectional studies, showed that PD patients had lower folate and vitamin B12 levels (SMD [95%CI]: −0.30[−0.39, −0.22], *p* < 0.001 for Vitamin B12; SMD [95%CI]: −0.20 [−0.28, −0.13], *p* < 0.001 for folate), but a significant higher Hcy level (SMD [95%CI]: 0.86 [0.59, 1.14], *p* < 0.001) than healthy people. Meanwhile, PD was significantly related to hyperhomocysteinemia (SMD [95%]: 2.02 [1.26, 2.78], *p* < 0.001) rather than plasma Hcy below 15 μmol/L (SMD [95%]: −0.31 [−0.62, 0.00], *p* = 0.05). Subgroup analysis revealed associations between the Hcy level of PD patients and region (*p* = 0.03), age (*p* = 0.03), levodopa therapy (*p* = 0.03), Hoehn and Yahr stage (*p* < 0.001), and cognitive impairment (*p* < 0.001). However, gender (*p* = 0.38) and sample size (*p* = 0.49) were not associated.

**Conclusion:**

Hcy, vitamin B12, and folic acid potentially predict the onset and development of PD. Additionally, multiple factors were linked to Hcy levels in PD patients. Further studies are needed to comprehend their roles in PD.

## 1. Introduction

Parkinson’s disease (PD) has become the second most common neurodegenerative disease in the elderly. The incidence and prevalence of PD rise with age in a steadily increasing manner (de Rijk et al., 1995; Pringsheim et al., 2014). As the global population ages, the prevalence of PD is projected to double over the next two decades (Dorsey et al., 2018a,b). It occurs rapidly worldwide without significant epidemiological differences between regions (Dorsey et al., 2018a; Bloem et al., 2021), but the number of patients is increasing rapidly in China, which accounted for more than a quarter of all patients in 2020 (Dorsey et al., 2018a,b; Qi et al., 2021).

PD is a chronic and incurable disease that is characterized by the presence of bradykinesia, muscular rigidity, resting tremor, and postural instability. It is also associated with various non-motor symptoms such as cognitive impairment, hyposmia, constipation, and mood disorders (Jankovic and Tan, 2020; Bloem et al., 2021). As a result, PD leads to a significant reduction in the quality of life of patients and their families with symptoms worsening over time (Hoogland et al., 2017). The main pathological changes of PD, the loss of dopaminergic neurons in the substantia nigra (SN) and the presence of Lewy bodies (LBs) and Lewy neurites (LNs; Dickson, 2018; Foffani and Obeso, 2018; Chen et al., 2020; Bloem et al., 2021), which consist of α-Synuclein (de Rijk et al., 1995; Hoogland et al., 2017), are universally acknowledged. However, the precise cause of this pathological change still remains unclear. Genes, lifestyle, aging, oxidative stress, and other factors are responsible for the degeneration and death of dopamine neurons (Martignoni et al., 2007; Chen et al., 2020; Jankovic and Tan, 2020; Bloem et al., 2021). Thus, the identification of treatable conditions that contribute to PD may reduce the burden on public health and the economy in a rapidly aging society (Fredericks et al., 2017).

**Figure 1:**
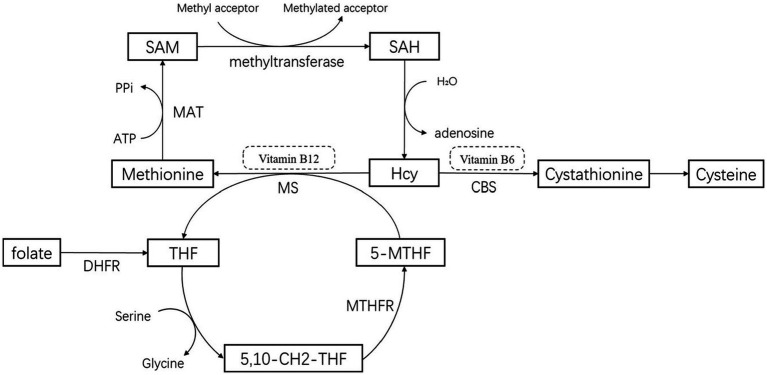
Pathways for the metabolism of homocysteine. 5-MTHF, 5-methyltetrahydrofolate; THF, Tetrahydrofolate; 5,10-CH2-THF, 5,10-methylenetetrahydrofolate; MTHFR, 5,10-methylenetetrahydrofolatereductase; CBS, Cystathionine-β -synthase; MS, methionine synthetase; MAT, methionine adenosyltransferase or S-adenosylmethionine synthetase; DHFR, Dihydrofolate Reductase.

Methionine, an essential amino acid derived from proteins in food, is metabolized in the erythrocytes to produce homocysteine (Hcy), a sulfur-containing amino acid. Homocysteine is primarily metabolized in the liver. The conversion of methionine to active methyl donor S-adenosylmethionine (SAM) occurs through the catalysis of S-adenosylmethionine synthetase, in which methionine combines with ATP. Subsequently, SAM is converted to S-adenosine homocysteine (SAH) by methyltransferase, resulting in the hydrolysis of Hcy and adenosine (Martignoni et al., 2007; Tinelli et al., 2019). The metabolism of Hcy involves two foremost pathways (de Rijk et al., 1995; Postuma and Lang, 2004; Martignoni et al., 2007; Sharma et al., 2015; Murray and Jadavji, 2019; Tinelli et al., 2019): The remethylation pathway, which converts Hcy to methionine, requires methionine synthetase (MS) and vitamin B12 as coenzymes. 5-Methyltetrahydrofolate (5-MTHF) supplies the methyl group for this pathway (Pringsheim et al., 2014); The sulfidation pathway is an irreversible reaction that requires cystathionine-β-synthetase (CBS) and vitamin B6 as coenzymes. It converts Hcy to cystathionine, which is then converted to cysteine (Figure 1). Based on the metabolism of Hcy, the enzyme MTHFR plays an important role in converting Hcy to methionine with vitamin B12 and folate as cofactors (Martignoni et al., 2007; Hu et al., 2013). Hence, it is hypothesized that the plasma levels of Hcy are negatively correlated with plasma folate and vitamin B12 levels.

Studies have shown that hyperhomocysteinemia (Hhcy), characterized by plasma Hcy levels exceeding 15 μmol/L, may be an independent risk factor for Parkinson’s disease (Martignoni et al., 2007; Licking et al., 2017; Xie et al., 2017; Li et al., 2020). PD patients have been found to have significantly higher Hcy levels compared to healthy individuals worldwide (Dong and Wu, 2020; Rahnemayan et al., 2022). Furthermore, Hcy levels in PD are associated with folate, vitamin B12, age, genetics, sex, L-dopa treatment, cognitive impairment, and so on (Hu et al., 2013; Collier et al., 2017). Both *in vitro* and *in vivo* studies have demonstrated that Hcy plays a role in the pathogenesis of mesencephalic dopaminergic neuronal death in PD (Wei et al., 2016).

However, the association between Hcy levels and the severity of PD has not been previously reported (Deng et al., 2022). In fact, previous studies have suggested that Hcy does not play a significant role in the pathogenesis of PD. For instance, studies by Rodriguez-Oroz and Camicioli found an unclear relationship between plasma Hcy levels and cognitive impairment in PD patients (Camicioli et al., 2009; Rodriguez-Oroz et al., 2009), contradicting the results of other studies (Song et al., 2013; Shen, 2015; Rahnemayan et al., 2022).

To sum up, the association between Hcy levels and PD remains controversial. Consequently, our study aimed to provide valuable insights and useful references for the clinical practice of PD, by systematically analyzing the association between plasma Hcy, folate, vitamin B12 levels, and PD.

## 2. Methods

### 2.1. Literature search

To conduct the meta-analysis, we followed the quality standards for such analysis. We searched the electronic databases PubMed, EMBASE, Medline, Ovid, and Web of Science to identify suitable studies published prior to May 2023 using the following search strategies (de Rijk et al., 1995): “Homocysteine” OR “Hcy” OR “Hyperhomocysteinemia” OR “Folate” OR “Folic acid” OR “Vitamin B9” OR “Vitamin B12” and (Pringsheim et al., 2014) “Parkinson” OR “Parkinson’s disease” OR “PD.” We only considered articles written in English, without any other restrictions. Moreover, we manually reviewed reference lists and citations to identify any additional relevant studies.

### 2.2. Inclusion and exclusion criteria

Studies were included if they met the following criteria:

1. The studies had to be original research, including population-based studies, case–control studies and cohort studies;
2. The patients included in the studies had to have a diagnosis of idiopathic Parkinson’s disease based on the UK Brain Bank criteria, with the exclusion of Parkinson’s syndrome, secondary Parkinson’s syndrome, and other psychopathies.
3. The controls in the studies had to be healthy individuals without a history of neurological disease or any disease related to Hcy, such as liver cirrhosis, end-stage renal disease, or malignancy;
4. The studies had to have received approval from the hospital ethics committee and informed consent was obtained from all patients;
5. The studies had to report complete information or provide enough data to calculate the mean and standard deviation of plasma Hcy, folate, or vitamin B12.

Studies were excluded if they met the following criteria:

1. Meta-analysis, review, case report, review, or letters;
2. Unable to obtain the full text;
3. Repeated or overlapped publications;
4. Data of the study was insufficient or undefined, including cohorts and data on the mean and standard deviation of plasma Hcy, folate, or vitamin B12.

### 2.3. Data extraction

The included studies were independently assessed by three reviewers. The extracted data included the name of the first author, publication year, the ethnicity of samples, the number of subjects, gender distribution, mean age, mean follow-up duration, mean and SD of Hcy, folate, and vitamin B12 levels for patients and controls, as well as the source and detection method used for Hcy, folate, and vitamin B12 analyses.

### 2.4. Quality evaluation

For the quality evaluation of the eligible studies, we used the Newcastle-Ottawa Quality Assessment Scale (NOS). The NOS utilizes a star system, allowing for a maximum score of nine stars spanning from 0 to 9. This system is divided into four parts: participant selection, comparability of the study group, exposure assessment, and outcome evaluation. A score below 7 indicates a low-quality study.

### 2.5. Statistical analysis

To explore the differences in Hcy, folate, and vitamin B12 levels between PD patients and control groups, we calculated the standard mean differences (SMD) accompanied by a 95% confidence interval (CI). If the cases were divided into different subgroups, we adopted the following method to integrate the data: $N=\overset{m}{\underset{i=1}{\sum }}N_{i}\text{;}$ $Mean_{T}\left(M_{T}\right)=\frac{\sum_{i=1}^{m}N_{i}M_{i}}{\sum_{i=1}^{m}N_{i}}\text{;}$ $SD=\sqrt{\frac{\sum_{i=1}^{m}\left(N_{i}-1\right){{SD}_{i}}^{2}+\sum_{i=1}^{m}N_{i}{\left(M_{i}-M_{T}\right)}^{2}}{\left(\sum_{i=1}^{m}N_{i}-1\right)}}$ . If the data was provided in the form of medians, extremes, or quartiles, we adopted Wan’s method (Wan et al., 2014) to convert it into the Mean and SD format.

Heterogeneity was evaluated using the Q-test and the I (Pringsheim et al., 2014)-test. The random-effects model was applied when I^2^ > 50%, indicating a high heterogeneity, while the fixed-effects model was applied otherwise. Moreover, subgroup analysis was conducted to identify the cause of heterogeneity. Subgroup analyses were stratified by sample size, Hcy concentration, cognitive impairment or dementia, gender, age, region, levodopa treatment, and Hoehn and Yahr stage. Sensitivity analysis was performed by removing each study one at a time to assess its impact on the result. Publication bias was assessed using Egger’s test, as it is a quantitative analysis. Statistical significance was defined as a two-sided *p*-value less than 0.05. All meta-analyses were performed by using R version 4.3.1.

## 3. Results

### 3.1. Basic characteristics and quality assessment of selected studies

**Figure 2:**
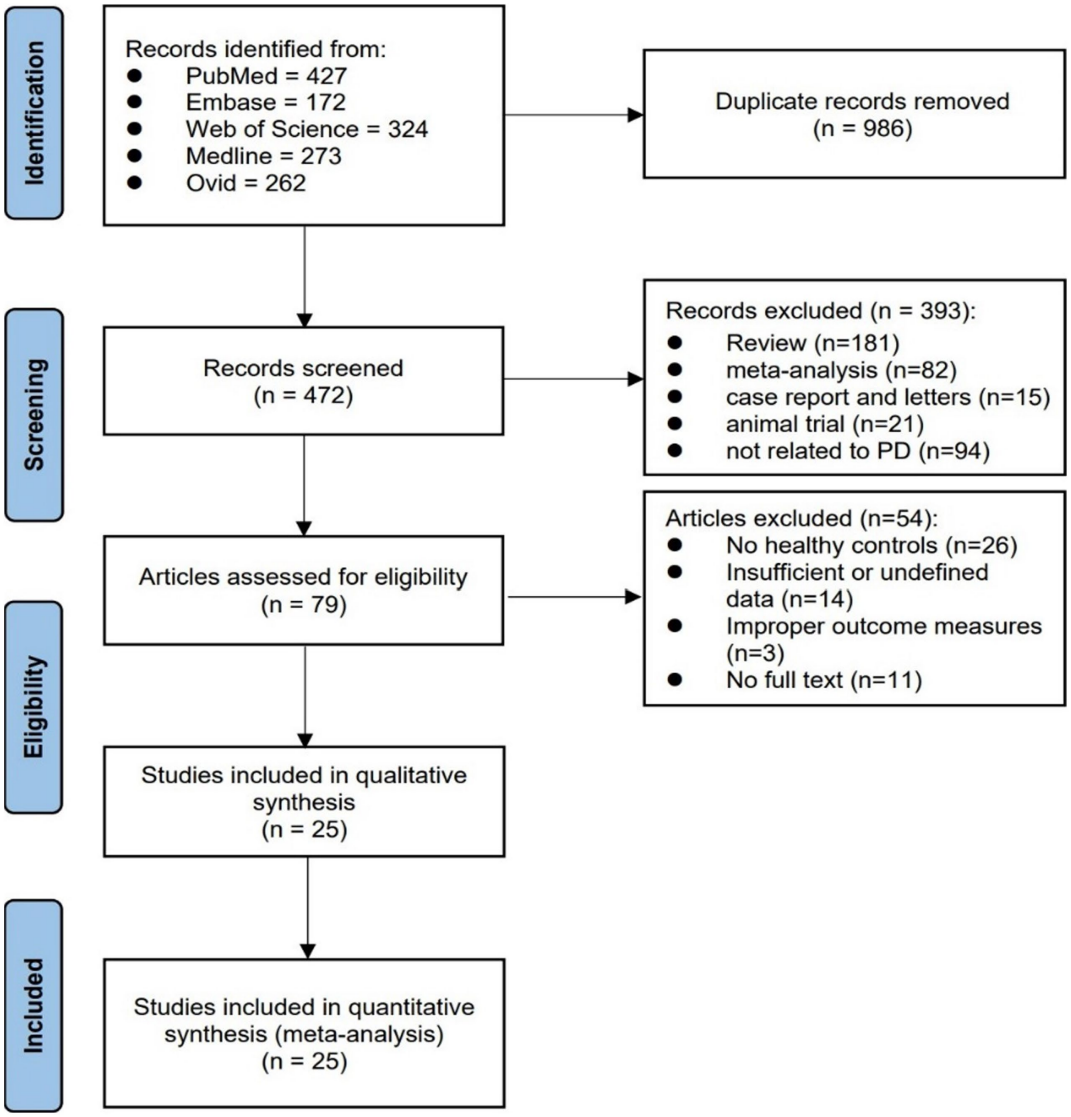
Flow diagram of the literature search and selection process.

A total of 1,458 articles were obtained in the initial search. After removing duplications, 438 articles remained. Subsequently, 359 articles were excluded based on the screening of titles and abstracts. Following this, a comprehensive analysis of the full texts of the 79 included articles was carried out, which included manually searching the citation and reference lists. Ultimately, 25 articles met the eligibility criteria and were eligible for the comprehensive analysis. Among these, 11 were case–control studies, 13 were cross-sectional studies, and 1 article included both a case–control study and a cross-sectional study. The selection flowchart is presented in Figure 2.

Among the 25 studies included in this review, the total number of participants was 4,689. Out of these participants, 2,359 were PD patients, while the remaining 2,330 were healthy controls. The included studies were published between 2004 and 2020 and were primarily conducted in Europe. The sample size varied from 60 to 574 across the included studies. Within these 25 studies, 23 studies provided data on the levels of Hcy, 16 studies provided data on folate and 14 studies provided data on vitamin B12. Furthermore, 5 studies specifically compared the Hcy concentrations between Parkinson’s disease dementia (PDD) and those without (nPDD). Additionally, 11 articles divided the PD patients into subgroups based on sex, age, Hcy levels, or drug therapy.

After evaluating the study quality, three studies scored 9, 12 studies scored 8, seven studies scored 7, two studies scored 6, and one study scored 5.

The basic characteristics and quality assessment results of the 25 included studies are presented below (Table 1). The overall results, original detailed data, and quality assessment results are illustrated in the Supplementary material.

**Table 1:** The baseline characteristics of the included studies.

No.	First author	Region	Study design	Sample size (male/female)		Age		Disease duration (year)	Duration of levodopa usage (year)	Score
				PD	Control group	PD	Control group			
1	Li et al. (2020)	China	case–control study	322 (186/136)	214 (108/106)	64.5 ± 8.5	63.8 ± 9.3	NA	NA	7
2	Chen et al. (2015)	China	cross-sectional study	60 (34/26)	50 (27/23)	63.1 ± 10.6	55.6 ± 10.8	NA	NA	8
3	Ozer et al. (2006)	turkey	cross-sectional study	39 (25/14)	28 (15/13)	67.0 ± 9.3	61.9 ± 8.3	6.4 ± 3.9	4.4 ± 3.5	8
4	Triantafyllou et al. (2008)	Athens	cross-sectional study	111 (65/46)	93 (NA)	70.1 ± 8.0	69.6 ± 8.1	5.9 ± 3.9	NA	7
5	Zoccolella et al. (2009)	Italy	cross-sectional study	121 (72/49)	154 (97/57)	67.8 ± 8.0	68.7 ± 8.8	NA	≥ 1-y	8
6	Zoccolella et al. (2005)	Italy	case–control study	45 (27/18)	15 (NA)	61.3 ± 9.0	61 ± 10.4	10.4 ± 6.2	NA	9
7	Lamberti et al. (2005)	Italy	case–control study	46 (33/13)	32 (22/10)	63.7 ± 8.9	64.5 ± 11.5	10.8 ± 5.0	7.2 ± 4.8	7
8	Triantafyllou et al. (2007)	Athens	case–control study	67 (37/30)	67 (NA)	69.9 ± 5.3	NA	7.3 ± 3.4	YES	6
9	Saadat et al. (2018)	Babol	cross-sectional and case–control study	100 (53/47)	100 (50/50)	NA	NA	NA	NA	5
10	Białecka et al. (2012)	Poland	case–control study	320 (164/156)	254 (136/118)	64.4 ± 10.1	64.8 ± 9.6	6.8 ± 5.2	YES	8
11	Ojo et al. (2011)	South western Nigeria	cross-sectional study	40 (32/8)	40 (32/8)	65.8 ± 9.8	63.3 ± 10.8	5.4 ± 0.81	NA	8
12	Caccamo et al. (2007)	Italy	case–control study	49 (22/27)	86 (40/46)	64.2 ± 7.5	64.1 ± 7.1	5.8 ± 4.1	4.8 ± 3.4	9
13	Camicioli et al. (2009)	Canada	cross-sectional study	51 (30/21)	50 (29/21)	71.5 ± 4.7	71.6 ± 4.9	8.74 ± 4.4	YES	8
14	Gorgone et al. (2012)	Italy	case–control study	60 (27/33)	82 (37/45)	64.5 ± 7.7	64.1 ± 7.2	NA	≥ 1-y	8
15	Lee et al. (2010)	Korea	cross-sectional study	96 (42/53)	285 (126/159)	67.6 ± 6.0	67.6 ± 6.0	5.7 ± 2.7	3.3 ± 1.7	7
16	Religa et al. (2006)	Poland	case–control study	114 (NA)	100 (NA)	70.0 ± 7.6	71.2 ± 6.0	5.5 ± 4.0	4.8 ± 10.4	9
17	Rodriguez-Oroz et al. (2009)	Spain	cross-sectional study	89 (52/37)	30 (16/14)	71.7 ± 6.4	68.5 ± 3.0	14.3 ± 4.4	YES	8
18	Sapkota et al. (2014)	Canada	cross-sectional study	46 (26/20)	49 (28/21)	70.8 ± 4.3	71.6 ± 4.0	8.42 ± 4.51	4.78 ± 4.18	8
19	Shin and Sohn (2009)	Korea	case–control study	33 (10/23)	41 (12/29)	63.5 ± 7.8	65.4 ± 7.8	≥ 3-y	≥ 3-y	8
20	Sławek et al. (2013)	Poland	cross-sectional study	192 (101/91)	184 (114/70)	63.7 ± 9.4	65.4 ± 9.2	6.8 ± 5.3	NA	8
21	Song et al. (2013)	Korea	cross-sectional study	61 (26/35)	48 (11/37)	68.3 ± 7.0	66.2 ± 11.8	3.0 ± 2.4	2.7 ± 2.5	7
22	Todorović et al. (2006)	Serbia	case–control study	113 (63/50)	53 (34/19)	61.1 ± 9.1	60.8 ± 13.1	3.1 ± 2.5	YES	7
23	Yuan et al. (2009)	China-Taiwan	case–control study	76 (28/48)	110 (37/73)	71.4 ± 9.8	69.9 ± 8.5	5.0 ± 4.0	6.2 ± 4.3	8
24	Wei et al. (2016)	China	cross-sectional study	17 (11/6)	85 (55/30)	71.0 ± 15.4	70.7 ± 12.1	NA	NA	6
25	Zou et al. (2018)	China	cross-sectional study	92 (49/47)	80 (44/36)	65.7 ± 11.2	64.4 ± 7.1	4.1 ± 3.4	YES	7

NA: not available.

### 3.2. Plasma folate and vitamin B12 levels in PD patients and controls

**Figure 3:**
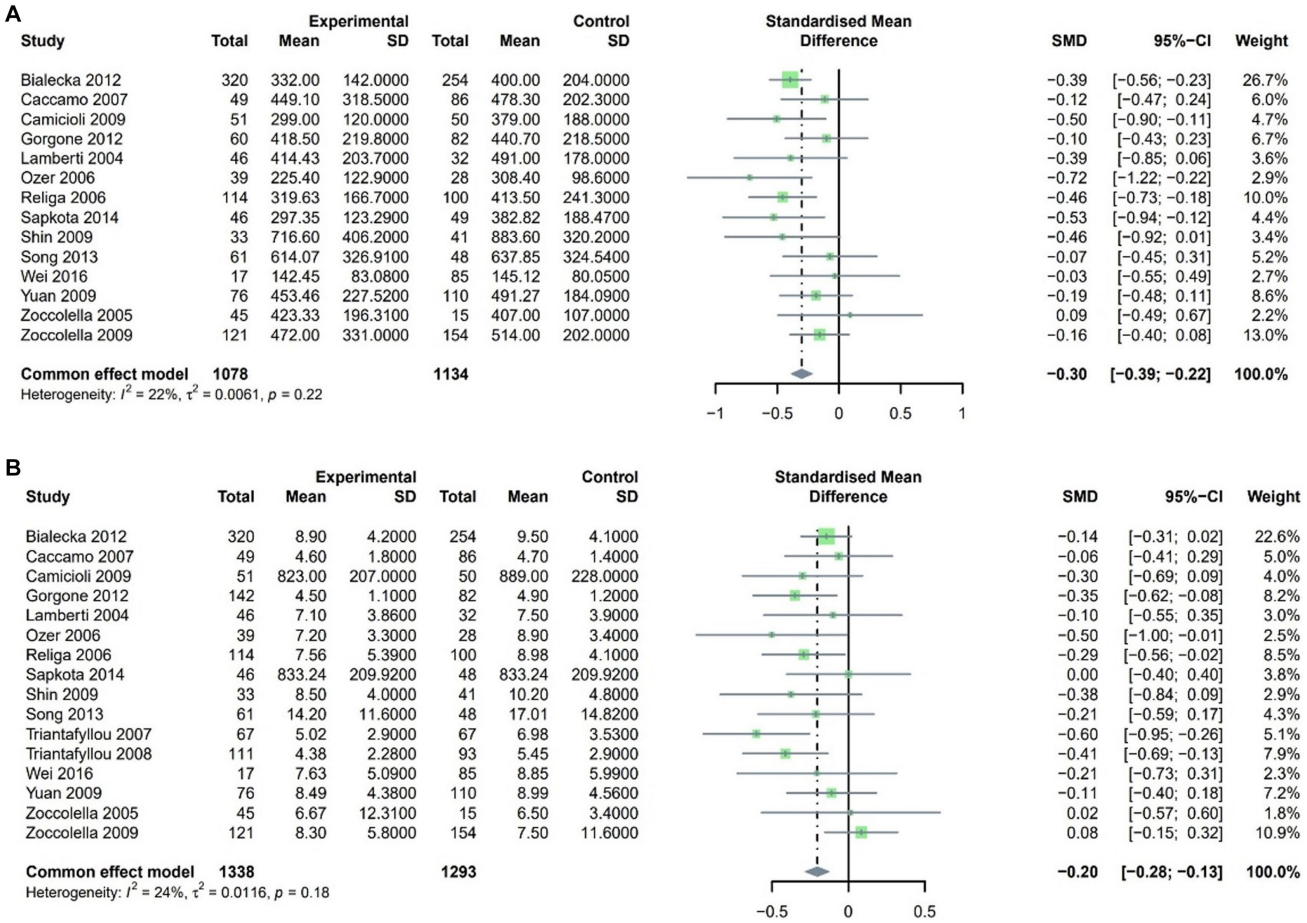
Forest plot standard mean difference (SMD) and 95% confidence interval (65%CI) in PD and control group for **(A)** VitaminB12 and **(B)** Folate.

We applied a fixed effect model as there was low heterogeneity for both Vitamin B12 and folate after pooling the included studies. According to the result, the levels of both Vitamin B12 and folate were lower in PD patients compared to controls. The SMD for vitamin B12 was-0.30 (95% CI [−0.39, −0.22], *p* < 0.001, I^2^ = 22%), and the SMD for folate was-0.20 (95% CI [−0.28, −0.13], *p* < 0.001, I^2^ = 24%; Figures 3A,B, respectively).

### 3.3. Plasma and serum Hcy levels in PD patients and controls

#### 3.3.1. General analysis

**Figure 4:**
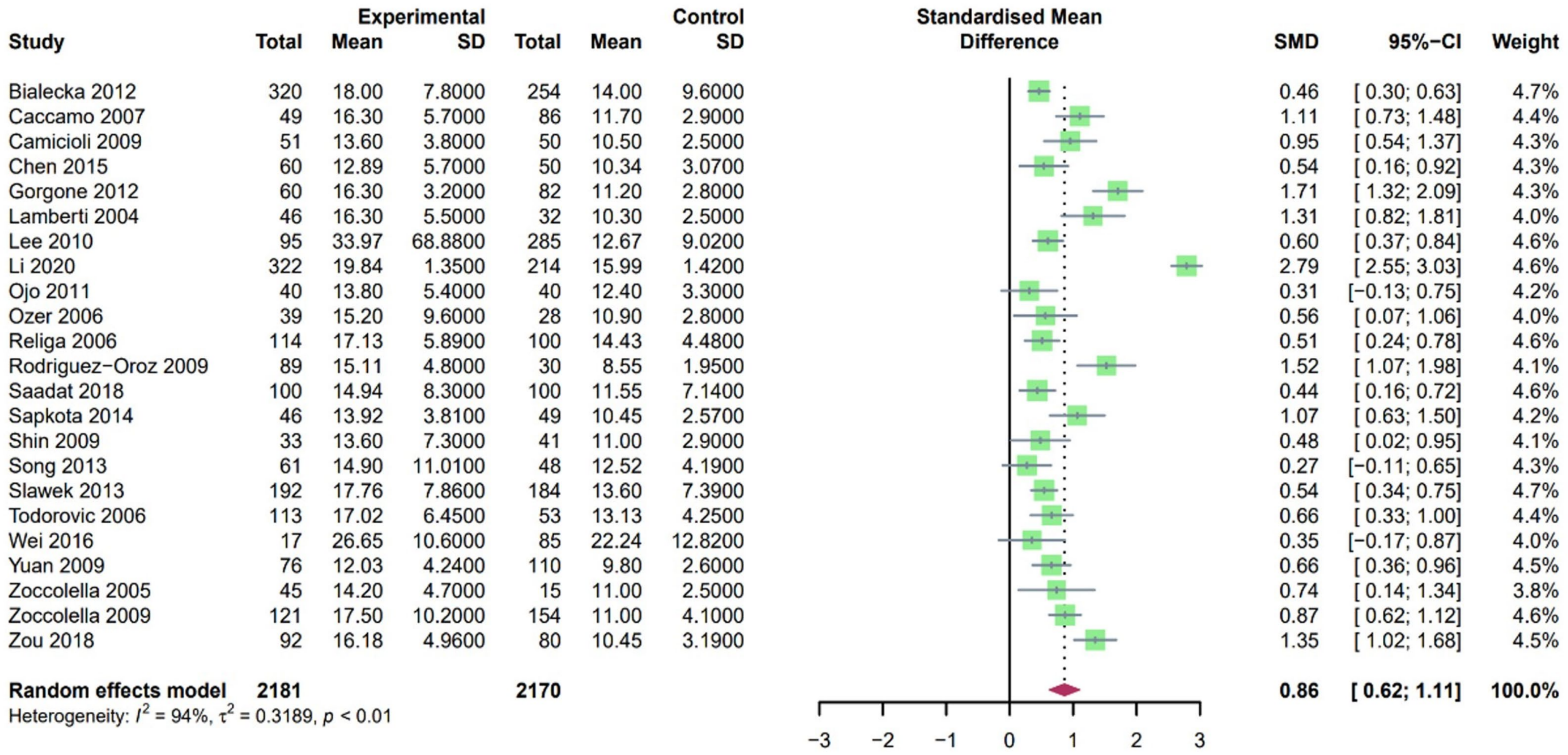
Forest plot standard mean difference (SMD) and 95% confidence interval (65%CI) in PD and control group for plasma homocysteine.

A random-effects model was employed due to the high heterogeneity found in the SMD for Hcy after pooling the included studies. Our findings revealed that plasma Hcy levels in PD patients were higher than those in controls, with an SMD of 0.86 (95% CI [0.59, 1.14], *p* < 0.001, I^2^ = 94%; Figure 4). Furthermore, sensitivity analysis confirmed that the results remained consistent even after the exclusion of any single study.

#### 3.3.2. Subgroups analyses

To investigate the impact of sample size, gender, average age, geographical ethnicity, Hcy concentration, Levodopa therapy, and cognitive and motor function on plasma Hcy levels in PD patients and controls, subgroup analyses were conducted (Table 2).

**Table 2:** Pooled SMD of plasma Hcy levels in PD patients and controls in subgroup analysis.

Subgroups		No. of studies	Sample size	SMD and 95% CI	*p* value	Test of heterogeneity *I^2^* (%)	Egger’s test for publication bias	
							t	*P* value
Sample size								
	<100	6	454	0.74 [0.43, 1.06]	<0.001	60	0.94	0.3476
	≥100	17	3,897	0.90 [0.57, 1.23]	<0.001	95	1.57	0.1175
Sex								
	Male*	3	307	0.61 [0.38, 0.66]	<0.001	0	−1.57	0.1172
	Female*	3	503	0.48 [0.30, 0.66]	<0.001	44	0.52	0.6015
Age								
	~60*	2	61	0.07 [−0.43, 0.57]	0.79	0	/	/
	60 ~ 70	18	3,439	0.82 [0.47, 1.18]	<0.001	95	0.80	0.4264
	70 ~ 80	8	962	0.92 [0.62, 1.22]	<0.001	75	−0.99	0.3223
	80~*	2	46	0.93 [0.32, 1.55]	0.003	0	/	/
Region								
	China	5	1,106	1.15 [0.14, 2.16]	0.03	98	−0.49	0.6242
	Korea*	3	563	0.51 [0.32, 0.69]	<0.001	6	−0.52	0.6015
	Europe	11	2,206	0.88 [0.65, 1.12]	<0.001	84	1.95	0.0516
Hhcy								
	Yes	3	139	2.02 [1.26, 2.78]	<0.001	63	3.00	0.1172
	No	3	182	−0.04 [−0.52, 0.60]	0.90	71	−0.52	0.6015
Levodopa therapy								
	Yes	19	3,280	0.87 [0.69, 1.06]	<0.001	82	1.85	0.0637
	NO*	4	536	0.41 [0.19, 0.62]	<0.001	14	0.00	1.0000
cognitive function								
	PDD or CI	6	1,103	1.11 [0.81, 1.40]	<0.001	74	1.32	0.1885
	No	5	1,108	0.55 [0.15, 0.95]	0.007	89	0.98	0.3272
H and Y								
	1 ~ 2	4	599	1.63 [−0.83, 4.10]	0.19	95	−0.68	0.4969
	2 ~ 3*	8	1,374	3.54 [3.07, 4.01]	<0.001	16	0.49	0.6207
	4 ~ 5	2	119	5.37 [3.20, 7.54]	<0.001	91	/	/

Hhcy, Hyperhomocysteinemia. PDD, Parkinson’s Disease with dementia. H and Y, Hoehn and Yahr Stage. * the fix-effect model was applied in the subgroup analysis.

Higher levels of Hcy in PD patients were observed in both subgroups based on sample size. In the subgroup analysis of a sample size of less than 100 participants, the SMD was 0.74 (95% CI: 0.43, 1.06, *p* < 0.001) with an I^2^ of 60%. Similarly, the subgroup analysis of a sample with 100 or more participants showed an SMD of 0.90 (95% CI: 0.57, 1.23, *p* < 0.001) with an I^2^ of 95%. When analyzing the data by gender, PD patients exhibited higher levels of Hcy in both males (SMD [95% CI]: 0.61 [0.38, 0.66], *p* < 0.001, I^2^ = 0%) and females (SMD [95% CI]: 0.48 [0.30, 0.66], *p* < 0.001, I^2^ = 44%). As for age, no significant difference in Hcy levels was observed in individuals aged younger than 60 years (SMD [95% CI]: 0.07 [−0.43, 0.57], *p* = 0.83, I^2^ = 0%), while a significant relationship was found in other age groups. Specifically, in the 60 ~ 70 age group, the SMD was 0.82 (95% CI: 0.47, 1.18, *p* < 0.001, I^2^ = 95%); in the 70 ~ 80 age group, the SMD was 0.92 (95% CI: 0.62, 1.22, *p* < 0.001, I^2^ = 75%); and in individuals aged 80 and older, the SMD was 0.93 (95% CI: 0.32, 1.55, *p* = 0.003, I^2^ = 0%). Furthermore, the results varied based on geographic regions. Significant differences in plasma Hcy levels between PD patients and controls were observed in subgroups from Korea (SMD [95% CI]: 0.51 [0.32, 0.91], *p* < 0.001, I^2^ = 6%), China (SMD [95% CI]: 1.15 [0.14, 2.16], *p* < 0.001, I^2^ = 98%), and Europe (SMD [95% CI]: 0.88 [0.65, 1.12], *p* < 0.001, I^2^ = 84%).

In the subgroup analysis, we found that high levels of Hcy played a significant role in PD patients compared with the controls (SMD [95% CI]: 2.02 [1.26, 2.78], *p* < 0.001, I^2^ = 63%). However, there was no statistically significant relationship between PD and Hcy levels below 15 μmol/L (SMD [95% CI]: −0.31 [−0.62, 0.00], *p* = 0.05, I^2^ = 0%). Additionally, the subgroup analysis based on levodopa therapy showed a significant difference (Yes, SMD [95% CI]: 0.87 [0.69, 1.06], *p* < 0.001, I^2^ = 82%; No, SMD [95% CI]: 0.41 [0.19, 0.62], *p* < 0.001, I^2^ = 14%). Similarly, the subgroup analysis based on cognitive function also revealed a significant difference (PDD or CI, SMD [95% CI]: 1.11 [0.81, 1.40], *p* < 0.001, I^2^ = 74%; No, SMD [95% CI]: 0.55 [0.15, 0.95], *p* < 0.001, I^2^ = 89%). Moreover, PD patients classified as Hoehn and Yahr Stage 3 ~ 5 had higher Hcy levels (H and Y Stage 2 ~ 3, SMD [95% CI]: 3.54 [3.07, 4.01], *p* < 0.001, I^2^ = 16%; H andY Stage 3 ~ 5, SMD [95% CI]: 5.37 [3.20, 7.54], *p* < 0.001, I^2^ = 91%), whereas PD patients with Hoehn and Yahr Stage 1 ~ 2 did not show a significant increase in Hcy levels (SMD [95% CI]: 1.63 [−0.83, 4.10], *p* = 0.19, I^2^ = 95%).

#### 3.3.3. Factors associated with Hcy in PD patients

The results of the included studies indicated low heterogeneity in Hcy levels and cognitive function (I^2^ < 50%, *p* > 0.05; Table 3). Therefore, the fixed-effects model was applied to analyze these two factors, while the random-effects model was utilized for the analysis of other factors. In line with these results, a significant relationship was observed between plasma Hcy levels and factors such as advanced age (70 and above) among PD patients, Hoehn and Yahr stage (Stage 3–5), cognitive impairment, and levodopa therapy. It was observed that PD patients within these categories exhibited slightly higher levels of Hcy.

**Table 3:** The influence of relevant factors on Hcy in PD patients in the meta-analysis.

Factors	Sample size		No. of studies	SMD [95% CI]	*P* value	Test of heterogeneity		Egger’s test for publication bias	
	case	control				*I* ^2^	P value	t	*p* value
Hhcy	38 (Yes)	69 (No)	3	10.82 [8.92, 12.71]	<0.001	0	0.90	1.57	0.1172
H and Y	96 (4 ~ 5)	156 (2 ~ 3)	1	0.45 [0.19,0.70]	<0.001	/	/	/	/
	96 (4 ~ 5)	61 (1 ~ 2)	1	0.46 [0.15,0.78]	<0.001	/	/	/	/
	315 (2 ~ 3)	61 (1 ~ 2)	4	0.34 [−0.04,0.72]	0.08	71	0.02	1.36	0.1742
Levodopa therapy	162 (Yes)	58 (No)	3	0.72 [0.08, 1.36]	0.03	71	0.03	0.52	0.6015
CI or dementia	221 (Yes)	438 (No)	5	0.54 [0.38, 0.71]	<0.001	7	0.37	−0.98	0.3272
Age	103 (70~)	129 (60 ~ 70)	3	0.58 [0.30, 0.85]	<0.001	0	0.82	0.52	0.6015
	24 (70~)	30 (~60)	2	0.84 [0.28, 1.41]	0.004	35	0.22	/	/
	45 (60 ~ 70)	14 (~60)	1	0.78 [0.16, 1.39]	0.01	/	/	/	/

H and Y, Hoehn and Yahr Stage; CI, cognitive impairment.

### 3.4. Sensitivity analysis and publication bias

To explore the potential sources of heterogeneity in the association between Hcy concentration and PD, a sensitivity analysis was conducted for all analyses. The influence of Hcy concentration on PD patients was examined through both fixed and random effects models. The stability of our meta-analysis results was confirmed as no significant changes were observed. Additionally, the absence of any significant publication bias was indicated by the results of Egger’s tests.

## 4. Discussion

The meta-analysis was a quantitative systematic analysis with 25 articles included. Drawing on this, we systematically analyzed the association between Hcy, folate, and vitamin B12 levels and the risk of PD. Our results confirmed that PD patients had significantly higher Hcy levels compared to the controls, as well as lower folic acid and vitamin B12 levels. These results of our meta-analysis were in accordance with most studies (Xie et al., 2017; Christine et al., 2018; Dong and Wu, 2020; Boelens Keun et al., 2021; Periñán et al., 2023). On the contrary, Wei et al.’s (2016) found no correlation between PD pathogenesis and high Hcy levels, nor did it find any association with vitamin B12 and folate deficiency. We think there are two reasons for this discrepancy. First, the study only included cases from Luliang City, a mountainous region in China, making the results non-representative of the wider population in China or globally. Second, Wei employed qualitative analysis instead of quantitative analysis to analyze plasma Hcy levels. Furthermore, the cases were divided into two subgroups using a cut-off of 22.175 𝜇mmol/L instead of the recommended 14 𝜇mmol/L serum Hcy, which could have significantly impacted the result.

Previous studies have shown that plasma folate and vitamin B12 levels are decreased in patients with Parkinson’s disease when compared to healthy individuals (Postuma and Lang, 2004; dos Santos et al., 2009; Jankovic and Tan, 2020). PD patients may experience reduced absorption of vitamin B12 and folate due to gastrointestinal dysfunction, a common nonmotor symptom of PD, accompanied by changes in gut microbiota (Chiang and Lin, 2019; Rosario et al., 2021). However, the treatment of PD patients with levodopa and catechol-O-methyltransferase inhibitors (COMT-I) may lower vitamin B12 and folate levels (Triantafyllou et al., 2007; Anamnart and Kitjarak, 2021), although the reasons for this effect remain unclear. Vitamin B12 provides protection against neuronal apoptosis by preventing DNA damage and is also a cofactor for two essential enzymes, methionine synthase, and methylmalonyl Coenzyme A mutase, involved in myelination. A deficiency in vitamin B12 also contributes to myelination of the peripheral and central nervous system (Moore et al., 2012; Christine et al., 2018). Similarly, folate acts as an antioxidant, reducing plasma Hcy levels and protecting DNA from damage (Martignoni et al., 2007; Murray and Jadavji, 2019). Additionally, studies have shown that older individuals with low blood concentrations of folate and vitamin B12 perform poorly on cognitive tests, such as tests of memory and nonverbal abstract thinking (Ajibawo-Aganbi et al., 2020). Supplementing with vitamin B12 and folate has been proven to significantly reduce vascular events and dementia (Ansari et al., 2014; Craenen et al., 2020; Anamnart and Kitjarak, 2021; Boelens Keun et al., 2021). However, a prospective, population-based cohort study suggested that dietary vitamin B6 may decrease the risk of PD, rather than folate and vitamin B12 (de Lau et al., 2006). Nonetheless, we still recommend the supplementation of vitamin B12 and folate in all PD patients as an adjuvant.

Furthermore, our study confirmed that hyperhomocysteine had a significant relationship with PD since a deficiency in vitamin B12 and folate leads to an increase in Hcy as they take part in the remethylation pathway of Hcy metabolism (Figure 1). Hhcy may play a crucial role in neurodegeneration in several ways. (1) High levels of Hcy induce neuronal apoptosis and worsen dopaminergic degeneration in PD patients through oxidative stress (Postuma and Lang, 2004; Martignoni et al., 2007), a clear cause and early feature of PD. Moreover, Hcy increases the vulnerability of dopaminergic neurons to oxidative stress damage (Postuma and Lang, 2004; Martignoni et al., 2007; Obeid et al., 2009). Hcy results in the generation of vast amounts of oxygen free radicals through two main mechanisms. First, the accumulation of SAH, a consequence of high Hcy, inhibits methyltransferases and increases catecholamine levels (Coppola et al., 2000), while decreasing the activity of antioxidant enzymes in cells such as SOD and catalase (Bhattacharjee and Borah, 2016). Second, high Hcy increases the activity of NADPH oxidase, further promoting oxidative stress (Takeno et al., 2016). (2) The dysfunction of ATP synthesis in mitochondria is related to Hcy, which leads to the death of neurons (Mattson and Shea, 2003; Jankovic & Tan, 2020). One possible mechanism for this dysfunction is the aggregation of α-synuclein (Zhou et al., 2023), a protein that regulates dopamine biosynthesis and homeostasis (Foffani and Obeso, 2018; Rocha et al., 2018), participates in endoplasmic reticulum/Golgi trafficking (Obeid et al., 2009), and is also influenced by Hcy (Zhou et al., 2023). (3) Hcy can directly damage neurons through nerve immune inflammation via glial cells (Postuma and Lang, 2004; Chen et al., 2015; Yan et al., 2018; Lee et al., 2019) and through a neurotoxic effect (Postuma and Lang, 2004; Martignoni et al., 2007; Murakami et al., 2010; Tinelli et al., 2019) that leads to cell death by increasing intracellular calcium levels (Foffani and Obeso, 2018). (4) It is commonly recognized that Hcy is a risk factor for cardiovascular disease as it damages vascular endothelial cells (Martignoni et al., 2007; Foffani and Obeso, 2018). Thus, Hcy may indirectly damage neurons by impacting the nutrition and blood supply to the nervous system, hastening the onset and progression of PD.

Subgroup analysis was conducted to investigate the factors contributing to the high heterogeneity in Hcy levels. While the results of subgroup analyses align with the overall analysis regarding the relationship between plasma Hcy level and PD, high heterogeneity remains apparent in certain subgroups.

Levodopa, the gold standard for the treatment of PD (Bloem et al., 2021), is acknowledged as an important factor contributing to an increase in plasma Hcy and SAM levels (Postuma and Lang, 2004; Lamberti et al., 2005; Hu et al., 2013). The methylation process of L-dopa to 3-O-methyldopa is catalyzed by catechol-O-methyltransferase (COMT), which consumes SAM as the methyl-group donor (Brosnan et al., 2004; Postuma and Lang, 2004; Lamberti et al., 2005; Zoccolella et al., 2009). Consequently, the levels of SAH, which can be readily hydrolyzed to Hcy, increase, ultimately leading to Hcy accumulation. Recognizing the association between elevated plasma Hcy and reduced methylation capacity, we conducted a subgroup analysis to evaluate the effect of levodopa therapy. The result showed that both levodopa-treated and non-levodopa-treated PD patients had higher Hcy levels compared to healthy individuals. Moreover, Hcy levels were higher in levodopa-treated PD patients compared to non-levodopa-treated PD patients. This indicates that both levodopa treatment and PD itself contribute to an increase in Hcy levels. Given that hyperhomocysteinemia is a well-established risk factor for multiple health complications, including cardiovascular disease and Alzheimer’s disease, we strongly recommend strict monitoring of the metabolic pathway components of levodopa in PD patients undergoing levodopa treatment.

Aging is confirmed as another main risk factor for nigrostriatal degeneration in several studies (Martignoni et al., 2007; Foffani and Obeso, 2018). No difference was found between Hcy and PD patients under the age of 60 in our research. The vulnerability of the brain region to oxidative insult in PD is attributed to the declining capacity of nigral dopamine neurons to offset ROS production with aging (Trist et al., 2019). Furthermore, a study discovered that α-syn within the cytoplasm of SN neuron cell bodies increases with aging (Collier et al., 2017), partly resulting in mitochondrial dysfunction and PD. Moreover, subgroup analysis also revealed varying concentrations of Hcy in PD patients of different ages. It is difficult to conclude that aging is a significant risk for high plasma Hcy, considering that older patients may have been receiving increasing doses of levodopa for many years and could have already developed PD. In summary, measuring plasma Hcy may be an alternative approach for predicting the onset and progression of PD in older individuals, particularly those aged 60 and above. Nonetheless, further research is necessary to establish critical values of Hcy for different age groups.

Cognitive impairment in PD is inevitable within 20 years of diagnosis, and up to 80% of PD patients develop dementia over the course of the disease (Hoogland et al., 2017). In addition to apoptosis, excitotoxicity, and oxidative stress, atherosclerosis resulting from high Hcy levels can also contribute to cognitive impairment (Postuma and Lang, 2004). The severity of motor symptoms in PD is measured using the Hoehn and Yahr Scale. Stages 1 and 2 represent a mild motor disorder that may be easily ignored by most patients. Stages 2 and 3 represent a moderate motor disorder that may affect the patient’s daily life. Stages 4 and 5 represent a severe motor disorder that prevents patients from living independently. We found that Hcy was also positively correlated with the severity of symptoms in PD, including cognitive and mobility impairment. Our results showed a significant increase in Hcy levels only in mild and advanced stages of PD. However, individual studies on the association between plasma Hcy level and the risk of dementia occurrence did not reach a consensus. There are four possible explanations for the conflicting results of Rodriguez-Oroz et al. (2009) and Camicioli et al. (2009), who found that high Hcy levels were not a risk factor for cognitive decline. First, their sample sizes were small, with only 89 and 51 PD patients respectively, which may not be representative of all PD patients. Second, they did not apply MMSE or PD-CRS to identify PDD, instead using Petersen’s criteria for Rodriguez-Oroz and the Dementia Rating Scale for Camicioli. Additionally, Camicioli focused on patients aged 71.5 whose Hcy levels may increase physiologically. Moreover, detailed data on Hcy in Rodriguez-Oroz’s study suggested a ceiling effect, indicating that the concentration of plasma Hcy might be lower than the actual levels. Hence, we still think that reducing plasma Hcy levels may be an effective approach to delaying disease progression. Although we observed significant differences in PD patients with cognitive impairment or mild/severe mobility impairment, we cannot solely attribute high plasma Hcy to the severity of PD due to the progressive nature of the disease and potential confounding factors such as levodopa usage. Nevertheless, Hcy could be a useful supplementary tool rather than a definitive method for assessing the severity of PD.

The risk of developing PD is twice as common in men than women (Dorsey et al., 2018a; Cerri et al., 2019), but we failed to find a significant difference in Hcy between genders. However, the SMD for Hcy in males was slightly higher than in females. It is well known that estradiol increases the synthesis, release, reuptake, and turnover of DA (Kompoliti, 2003). Additionally, estradiol has anti-inflammatory properties that protect neurons by attenuating microglia activation and modulating microglia polarization toward a cytoprotective phenotype (Cerri et al., 2019; Bustamante-Barrientos et al., 2021). However, estrogen and its derivatives decline in women after menopause, which typically occurs around 50 years old. Since the average age of patients included in our study was mostly over 60, the age at which the protective effect of estradiol appears to diminish in women, our study found no apparent difference between males and females.

A dramatic variation in Hcy levels among PD patients was observed across different regions. One possible explanation for this difference is the influence of lifestyle. Previous studies have indicated that an increased consumption of dairy products and alcohol is associated with high Hcy levels in PD patients (Ishihara and Brayne, 2005; Park et al., 2005; Jankovic and Tan, 2020). Conversely, some studies have suggested that the intake of tea or coffee and smoking may potentially decrease the risk of PD. (Ishihara and Brayne, 2005; Li et al., 2015; Ascherio and Schwarzschild, 2016) Furthermore, there is evidence to suggest that exercise is related to a reduction in Hcy levels in PD patients (Nascimento et al., 2011; Yang et al., 2015).

## 5. Limitations and strengths

This study had several limitations. First, only studies published in English were included in the analysis. Second, our meta-analysis did not include any prospective research that proves Hcy is a risk factor for PD. Moreover, the studies included in our analysis were predominantly conducted in East-Asian and European countries. The reason for the lack of American studies is that the research from America we found primarily focused on PD patients and lacked data on healthy individuals (Licking et al., 2017). In addition, it is worth noting that although some studies indicated a relationship between Hcy and other factors such as genetics (Postuma and Lang, 2004; Todorović et al., 2006; Zhu et al., 2015), types of PD (Christine et al., 2018; Shen et al., 2019), depression (Zhang et al., 2023), and minor hallucinations (Zhong et al., 2022), we did not analyze these connections in our study.

Our investigation included all studies that provided detailed raw data on Hcy, vitamin B12, and folate levels in PD and healthy individuals. To ensure comprehensiveness, we conducted thorough searches of five databases. To mitigate the effect of different units and measurement methods, we employed SMD to estimate the difference between PD patients and controls. Subgroup, sensitivity, and bias analyses were performed to identify potential sources of high heterogeneity. As a result, our results can be considered reliable and precise.

## 6. Conclusion

In summary, PD patients had lower folate and vitamin B12 levels, but significantly higher Hcy levels. These findings have implications for the treatment of PD in patients. Although hyperhomocysteinemia is not specific to PD, the measured Hcy can be seen as a surrogate marker of vitamin B12 and folate deficiency. Nonetheless, further in-depth research is warranted to confirm if Hcy can predict the onset and estimate the progression of PD, as well as to determine if it can be targeted for treatment.

## Data availability statement

The datasets presented in this study can be found in online repositories. The names of the repository/repositories and accession number(s) can be found in the article/Supplementary material.

## Author contributions

YQ: Conceptualization, Data curation, Formal analysis, Methodology, Validation, Visualization, Writing–original–draft, Writing – review & editing. ZG: Data curation, Formal analysis, Methodology, Visualization, Writing – review & editing. QX: Data curation, Formal analysis, Visualization, Writing – review & editing. JX: Data curation, Formal analysis, Visualization, Writing – review & editing. RO: Formal analysis, Methodology, Visualization, Writing – review & editing. HS: Formal analysis, Methodology, Project administration, Supervision, Writing – review & editing. QW: Conceptualization, Data curation, Formal analysis, Methodology, Writing–original–draft, Writing – review & editing.
