# Supplementary material for: Association between the risk and severity of Parkinson’s disease and plasma homocysteine, vitamin B12 and folate levels: a systematic review and meta-analysis
Source: Front Aging Neurosci. 2023 Oct 24;15:1254824. doi: 10.3389/fnagi.2023.1254824 (PMC10628521; doi:10.3389/fnagi.2023.1254824)
Supplement: Supplementary file 1 [file Data_Sheet_1.docx]

Supplementary Material

**Table S1.** The baseline characteristics of studies included in the meta-analysis.

| **ID** | **First author** | **Article title** | **Published year** | **Study design** | **Country** | **Inclusion period** | **Sample size (total)** | **Subgroups** | **Experimental groups** | | | | | **Control group** | | |
| --- | --- | --- | --- | --- | --- | --- | --- | --- | --- | --- | --- | --- | --- | --- | --- | --- |
|  |  |  |  |  |  |  |  |  | **n** | **Age at baseline (years)** | **Gender (M/F)** | **Disease duration（year）** | **Duration of levodopa usage** | **n** | **Age at baseline (years)** | **Gender (M/F)** |
| 1 | Li^1^ | Correlations between blood lipid, serum cystatin C, and homocysteine levels in patients with Parkinson’s disease | 2020 | case–control study | China | October 2014 to October 2016 | 536 | Total | 332 | 64.47 ±8.54 | 186/136 | NA | NA | 214 | 63.84 ±9.32 | 108/106 |
| 2 | Chen^2^ | Contra-Directional Expression of Serum Homocysteine and Uric Acid as Important Biomarkers of Multiple System Atrophy Severity: A Cross-Sectional Study | 2015 | cross-sectional study | China | July 2011 to November 2014 | 110 | Total | 60 | 63.10 ±10.62 | 34/26 | NA | NA | 50 | 55.64 ±10.82 | 27/23 |
| 3 | Ozer^3^ | Plasma homocysteine levels in patients treated with levodopa: motor and cognitive associations | 2006 | cross-sectional study | turkey | NA | 67 | Total | 39 | 67.0 ±9.3 | 25/14 | 6.4±3.9 | 4.4±3.5 | 28 | 61.9 ±8.3 | 15/13 |
|  |  |  |  |  |  |  |  | Hcy level ＞14 μmol/L | 17 | 68.5 ±8.8 |  | 6.4±3.3 |  | 20 | 61.9 ±9.1 |  |
|  |  |  |  |  |  |  |  | Hcy level ＜ 14 μmol/L | 17 | 64.4 ±9.5 |  | 6.4±4.3 |  | 20 |  |  |
| 4 | Triantafyllou^4^ | Folate and vitamin B12 levels in levodopa-treated Parkinson’s disease patients: Their relationship to clinical manifestations, mood and cognition | 2008 | cross-sectional study | Athens | NA | 204 | Total | 111 | 70.1 ±8.0 | 65/46 | 5.9±3.9 |  | 93 | 69.6 ±8.1 |  |
|  |  |  |  |  |  |  |  | Male | 65 | 69.0 ±8.6 |  | 6.1±3.4 |  |  |  |  |
|  |  |  |  |  |  |  |  | Female | 46 | 71.6 ±7.1 |  | 5.9±4.6 |  |  |  |  |
| 5 | Zoccolella^5^ | Hyperhomocysteinemia in levodopa-treated patients with Parkinson's disease dementia | 2009 | cross-sectional study | Italy | NA | 275 | Total  (L-Dopa treated) | 121 | 67.8 ±8.0 | 72/49 |  | ＞1-year | 154 | 68.7 ±8.8 | 97/57 |
|  |  |  |  |  |  |  |  | PDD | 42 | 71.2 ±6.3 | 27/15 | 10±5.7 |  |  |  |  |
|  |  |  |  |  |  |  |  | nPDD | 79 | 65.4 ±8.0 | 45/34 | 10±6.4 |  |  |  |  |
| 6 | Zoccolella^6^ | Plasma homocysteine levels in Parkinson's disease: role of antiparkinsonian medications | 2005 | cross-sectional study | Italy | NA | 60 | Dopamine-agonists treated | 15 | 60.7 ±8.4 | 9/6 | 4.7±3.6 |  | 15 | 61.0 ±10.4 |  |
|  |  |  |  |  |  |  |  | L-Dopa treated | 15 | 61.9 ±8.1 | 9/6 | 9.8±4.8 |  |  |  |  |
|  |  |  |  |  |  |  |  | L-Dopa and COMT-I treated | 15 | 61.3 ±10.2 | 9/6 | 13.5±5.8 |  |  |  |  |
| 7 | Lamberti^7^ | Effects of Levodopa and COMT Inhibitors on Plasma Homocysteine in Parkinson’s Disease Patients | 2004 | cross-sectional study | Italy | NA | 78 | L-Dopa treated | 26 | 64.0 ±8.5 | 19/7 | 9.3±4.2 | 5.3±3.6 | 32 | 64.5 ±11.5 | 22/10 |
|  |  |  |  |  |  |  |  | L-Dopa and COMT-I treated | 20 | 63.3 ±9.6 | 14/6 | 12.8±5.4 | 9.6±5.1 |  |  |  |
| 8 | Triantafyllou^8^ | The Influence of Levodopa and the COMT Inhibitor on Serum Vitamin B12 and Folate Levels in Parkinson’s Disease Patients | 2007 | case–control study | Athens | NA | 134 | Total | 67 | 69.9 ±5.3 | 37/30 | 7.3±3.4 |  | 67 |  |  |
| 9 | Saadat^9^ | Serum Homocysteine Level in Parkinson's Disease and Its Association with Duration, Cardinal Manifestation, and Severity of Disease | 2018 | cross-sectional and case-control study | Babol | 2015 to 2016 | 200 | Total | 100 |  | 53/47 |  |  | 100 |  | 50/50 |
| 10 | Biaecka^10^ | Association of COMT, MTHFR, and SLC19A1(RFC-1) polymorphisms with homocysteine blood levels and cognitive impairment in Parkinson’s disease | 2012 | case–control study | Poland | January 2008 to December 2010 | 574 | Total | 320 | 64.4 ±10.1 | 164/156 | 6.8±5.2 |  | 254 | 64.8 ±9.6 | 136/118 |
|  |  |  |  |  |  |  |  | nPDD | 153 | 62.1 ±9.7 | 78/75 | 6.0±4.7 |  |  |  |  |
|  |  |  |  |  |  |  |  | PDD | 64 | 68.8 ±9.3 | 34/30 | 8.3±5.6 |  |  |  |  |
| 11 | Ojo^11^ | Plasma homocysteine level and its relationship to clinical profile in Parkinson's disease patients at the Lagos University Teaching Hospital | 2011 | case-control design | South western Nigeria | March to September 2006 | 80 | Total | 40 | 65.8 ±9.8 | 32/8 | 64.9±9.8 (month) | 5.4±0.81 | 40 | 63.3 ±10.8 | 32/8 |
|  |  |  |  |  |  |  |  | Hcy level ＞14 μmol/L | 9 | 69.7 ±7.3 |  | 88±53.3 |  |  |  |  |
|  |  |  |  |  |  |  |  | Hcy level ＜ 14 μmol/L | 31 | 64.9 ±10.2 |  | 58.1±47.2 |  |  |  |  |
| 12 | Caccamo^12^ | Effect of MTHFR Polymorphisms on Hyperhomocysteinemia in Levodopa-treated Parkinsonian Patients | 2007 | case–control study | Italy | NA | 135 | Total | 49 | 64.2 ±7.5 | 22/27 | 5.8±4.1 | 4.8±3.4 | 86 | 64.1 ±7.1 | 40/46 |
| 13 | Camicioli^13^ | Homocysteine Is Not Associated with Global Motor or Cognitive Measures in Nondemented Older Parkinson’s Disease Patients | 2009 | cross-sectional and case-control study | Canada | NA | 101 | Total | 51 | 71.5 ±4.7 | 30/21 | 8.74±4.4 |  | 50 | 71.6 ±4.9 | 29/21 |
| 14 | Gorgone^14^ | Coenzyme Q10, Hyperhomocysteinemia and MTHFR C677T Polymorphism in Levodopa-treated Parkinson’s Disease Patients | 2012 | case–control study | Italy | NA | 142 | Total | 60 | 64.5 ±7.7 | 27/33 |  | ＞1-year | 82 | 64.1 ±7.2 | 37/45 |
| 15 | Lee^15^ | Hyperhomocysteinemia Due to Levodopa Treatment as a Risk Factor for Osteoporosis in Patients with Parkinson’s Disease | 2010 | cross-sectional and case-control study | Korea | October 2006 to May 2007 | 380 | Total | 95 | 67.6 ±6.0 | 42/53 | 39 (0–195)median |  | 285 | 67.6 ±6.0 | 126/159 |
| 16 | Religa^16^ | Hyperhomocysteinemia and methylenetetrahydrofolate reductase polymorphism in patients with Parkinson's disease | 2006 | case–control study | Poland | NA | 214 | L-Dopa treated | 99 | 70.5 ±7.57 |  | 6.06±4.05 | 4.8±10.35 | 100 | 71.2 ±6.0 |  |
|  |  |  |  |  |  |  |  | not L-Dopa treated | 15 | 66.0 ±7.11 |  | 1.97±1.02 |  |  |  |  |
| 17 | Rodriguez-Oroz^17^ | Homocysteine and cognitive impairment in Parkinson's disease: a biochemical, neuroimaging, and genetic study | 2009 | cross-sectional and case-control study | Spain | NA | 119 | PD-CN | 37 | 69.97 ±6.5 | 20/17 | 14.68±4.62 |  | 30 | 68.48 ±2.98 | 16/14 |
|  |  |  |  |  |  |  |  | PD-MCI | 22 | 70.23 ±5.2 | 14/8 | 13.05±3.69 |  |  |  |  |
|  |  |  |  |  |  |  |  | PDD | 30 | 74.87 ±6.15 | 18/12 | 14.73±4.45 |  |  |  |  |
| 18 | Sapkota^18^ | Association of homocysteine with ventricular dilatation and brain atrophy in Parkinson's disease | 2014 | cross-sectional and case-control study | Canada | April 2003 to September 2009 | 95 | Total | 46 | 70.97 ±4.26 | 26/20 | 8.42±4.51 | 4.78±4.18 | 49 | 71.63 ±4.80 | 28/21 |
| 19 | Shin^19^ | Hyperhomocysteinemia in patients with Parkinson's disease and relationship to vitamin B level | 2009 | case–control study | Korea | NA | 74 | L-Dopa treated | 33 | 63.5 ±7.8 | 10/23 | ＞3-years |  | 41 | 65.4 ±7.8 | 12/29 |
| 20 | Slawek^20^ | The impact of MRI white matter hyperintensities on dementia in Parkinson's disease in relation to the homocysteine level and other vascular risk factors | 2013 | cross-sectional and case-control study | Poland | January 2008 to December 2010 | 376 | Total | 192 | 63.7 ±9.4 | 101/91 | 6.8±5.3 |  | 184 | 65.35 ±9.16 | 114/70 |
|  |  |  |  |  |  |  |  | nPDD | 135 | 61.91 ±9.09 | 73/62 | 5.98±4.66 |  |  |  |  |
|  |  |  |  |  |  |  |  | PDD | 57 | 67.98 ±8.79 | 28/29 | 8.71±6.32 |  |  |  |  |
| 21 | Song^21^ | Clinical significance of homocysteine (hcy) on dementia in Parkinson's disease (PD) | 2013 | cross-sectional and case-control study | Korea | October 2010 to December 2011 | 109 | nPDD | 33 | 66.45 ±6.60 | 14/19 | 29.12±12.01 | 25.12±12.01 (month) | 48 | 66.23 ±11.83 | 11/37 |
|  |  |  |  |  |  |  |  | PDD | 28 | 70.50 ±6.75 | 12/16 | 44.01±39.998 | 41.51±40.09 (month) |  |  |  |
| 22 | Todorović^22^ | Homocysteine serum levels and MTHFR C677T genotype in patients with Parkinson's disease, with and without levodopa therapy | 2006 | case–control study | Serbia | NA | 166 | not L-Dopa treated | 30 | 59.13 ±8.65 | 18/12 | 1.69 ± 0.59 |  | 53 | 60.83 ±13.13 | 34/19 |
|  |  |  |  |  |  |  |  | L-Dopa treated | 83 | 61.86 ±9.14 | 45/38 | 3.70 ± 2.70 |  |  |  |  |
| 23 | Yuan^23^ | Methylenetetrahydrofolate reductase polymorphisms and plasma homocysteine in levodopa-treated and non-treated Parkinson's disease patients | 2009 | case–control study | China-Taiwan | NA | 186 | L-Dopa treated | 48 | 71.83 ±10.34 | 17/31 | 6.56±4.30 | 6.23±4.33 | 110 | 69.95 ±8.46 | 37/73 |
|  |  |  |  |  |  |  |  | not L-Dopa treated | 28 | 70.57 ±9.09 | 11/17 | 2.45±1.37 |  |  |  |  |
| 24 | Wei^24^ | Parkinson's Disease and Homocysteine: A Community-Based Study in a Folate and Vitamin B12 Deficient Population | 2016 | cross-sectional and case-control study | China | NA | 102 | Total | 17 | 71.0 ±15.36 | 11/6 |  |  | 85 | 70.7 ±12.1 |  |
| 25 | Zou^25^ | Trefoil Factor 3, Cholinesterase and Homocysteine: Potential Predictors for Parkinson's Disease Dementia and Vascular Parkinsonism Dementia in Advanced Stage | 2018 | cross-sectional and case-control study | China | November 2013 to October 2016 | 172 | PDD | 92 | 65.73 ±11.18 | 49/47 | 4.05±3.40 |  | 80 | 64.43 ±7.10 | 55/30 |
| NA: not available; ; CN: cognitively normal; MCI: mild cognitive impairment; PDD: PD patients with dementia. | | | | | | | | | | | | | | | | |

**Table S2.** The quality assessment using the Newcastle-Ottawa Scale for the included studies.

| **ID** | **Study** | **selection** | | | | **Comparability of cases and controls on the basis of the design or analysis** | **Exposure** | | | **Total score** |
| --- | --- | --- | --- | --- | --- | --- | --- | --- | --- | --- |
|  |  | **Is the case definition adequate** | **Representativeness of the cases** | **Selection of Controls** | **Definition of Controls** |  | **Ascertainment of exposure** | **Same method of ascertainment for cases and controls** | **Non-Response rate** |  |
| 1 | Li 2020 | √ | √ |  | √ | √ | √ | √ | √ | 7 |
| 2 | Chen 2015 | √ | √ |  | √ | √√ | √ | √ | √ | 8 |
| 3 | Ozer 2006 | √ | √ |  | √ | √√ | √ | √ | √ | 8 |
| 4 | Triantafyllou 2008 | √ | √ |  |  | √√ | √ | √ | √ | 7 |
| 5 | Zoccolella 2009 | √ | √ | √ | √ | √ | √ | √ | √ | 8 |
| 6 | Zoccolella 2005 | √ | √ | √ | √ | √√ | √ | √ | √ | 9 |
| 7 | Lamberti 2004 | √ | √ |  | √ | √ | √ | √ | √ | 7 |
| 8 | Triantafyllou 2007 | √ | √ |  |  | √ | √ | √ | √ | 6 |
| 9 | Saadat 2018 | √ |  |  | √ |  | √ | √ | √ | 5 |
| 10 | Biaecka 2012 | √ | √ | √ | √ | √ | √ | √ | √ | 8 |
| 11 | Ojo 2011 | √ | √ | √ | √ | √ | √ | √ | √ | 8 |
| 12 | Caccamo 2007 | √ | √ | √ | √ | √√ | √ | √ | √ | 9 |
| 13 | Camicioli 2009 | √ | √ | √ | √ | √ | √ | √ | √ | 8 |
| 14 | Gorgone 2012 | √ | √ | √ | √ | √ | √ | √ | √ | 8 |
| 15 | Lee 2010 | √ | √ |  | √ | √ | √ | √ | √ | 7 |
| 16 | Religa 2006 | √ | √ | √ | √ | √√ | √ | √ | √ | 9 |
| 17 | Rodriguez-Oroz 2009 | √ | √ | √ | √ | √ | √ | √ | √ | 8 |
| 18 | Sapkota 2014 | √ | √ | √ | √ | √ | √ | √ | √ | 8 |
| 19 | Shin 2009 | √ | √ |  | √ | √√ | √ | √ | √ | 8 |
| 20 | Slawek 2013 | √ | √ | √ | √ | √ | √ | √ | √ | 9 |
| 21 | Song 2013 | √ | √ |  | √ | √ | √ | √ | √ | 7 |
| 22 | Todorović 2006 | √ | √ |  | √ | √ | √ | √ | √ | 7 |
| 23 | Yuan 2009 | √ | √ |  | √ | √√ | √ | √ | √ | 8 |
| 24 | Wei 2016 | √ |  | √ |  | √ | √ | √ | √ | 6 |
| 25 | Zou 2018 | √ | √ |  | √ | √ | √ | √ | √ | 7 |

**Table S3.** Characteristics of Hcy (μmol/L) of included studies.

| **ID** | **Study** | **Sample size** | **Source of the sample** | **Method** | **Subgroups** | **Experimental groups** | | | **Control group** | | |  |
| --- | --- | --- | --- | --- | --- | --- | --- | --- | --- | --- | --- | --- |
|  |  |  |  |  |  | **n** | **MEAN** | **SD** | **n** | **MEAN** | **SD** |  |
| 1 | Li 2020 | 536 | NA | NA | Total | 322 | 19.84 | 1.35 | 214 | 15.99 | 1.42 |  |
|  |  |  |  |  | H and Y 1-2 | 70 | 19.59 | 1.35 |  |  |  |  |
|  |  |  |  |  | H and Y 3-4 | 156 | 19.65 | 1.36 |  |  |  |  |
|  |  |  |  |  | H and Y 5 | 96 | 20.33 | 1.74 |  |  |  |  |
| 2 | Chen 2015 | 110 | serum | SPCEI | Total | 60 | 12.89 | 5.7 | 50 | 10.34 | 3.07 |  |
| 3 | Ozer 2006 | 67 | NA | FPIA | Total | 39 | 15.20 | 9.60 | 28 | 10.90 | 2.80 |  |
|  |  |  |  |  | Hcy level ＞ 14 μmol/L | 17 | 21.9 | 11.3 | 20 | 10 | 2.8 |  |
|  |  |  |  |  | Hcy level ＜ 14 μmol/L | 17 | 10.1 | 2.6 | 20 | 10 | 2.8 |  |
| 5 | Zoccolella 2009 | 275 | plasma | HPLC | Total (L-Dopa treated) | 121 | 17.5 | 10.2 | 154 | 11 | 4.1 |  |
|  |  |  |  |  | PDD | 42 | 20.7 | 12.1 |  |  |  |  |
|  |  |  |  |  | nPDD | 79 | 15.8 | 8.5 |  |  |  |  |
| 6 | Zoccolella 2005 | 70 | plasma | HPLC | Dopamine-agonists treated | 15 | 12.4 | 2.2 | 15 | 11 | 2.5 |  |
|  |  |  |  |  | L-Dopa treated | 15 | 18.9 | 5.9 |  |  |  |  |
|  |  |  |  |  | L-Dopa and COMT-I treated | 15 | 12.8 | 3.4 |  |  |  |  |
| 7 | Lamberti 2004 | 78 | plasma | HPLC | L-Dopa treated | 26 | 17.7 | 6.1 | 32 | 10.3 | 2.5 |  |
|  |  |  |  |  | L-Dopa and COMT-I treated | 20 | 14.4 | 4.2 |  |  |  |  |
| 9 | Saadat 2018 | 200 | serum | ELISA method | male | 53 | 14.73 | 6.46 |  | 11.87 | 2.72 |  |
|  |  |  |  |  | female | 47 | 15.17 | 10.05 |  | 11.18 | 10.05 |  |
|  |  |  |  |  | ＜60 | 16 | 12.3 | 3.21 |  | 12.01 | 0.52 |  |
|  |  |  |  |  | 60-80 | 72 | 15.54 | 8.2 |  | 11.78 | 2.87 |  |
|  |  |  |  |  | ＞80 | 12 | 14.79 | 6.02 |  | 11.18 | 2.15 |  |
| 10 | Białecka 2012 | 574 | plasma | FPIA | Total | 320 | 18 | 7.8 | 254 | 14 | 9.6 |  |
|  |  |  |  |  | nPDD | 153 | 16.5 | 6.3 |  |  |  |  |
|  |  |  |  |  | PDD | 64 | 21.3 | 9 |  |  |  |  |
| 11 | Ojo 2011 | 80 | plasma | FPIA | Total | 40 | 13.8 | 5.4 | 40 | 12.4 | 3.3 |  |
|  |  |  |  |  | Hcy level ＞ 14 μmol/L | 9 | 21.9 | 3 |  |  |  |  |
|  |  |  |  |  | Hcy level ＜ 14 μmol/L | 31 | 11.1 | 2.9 |  |  |  |  |
| 12 | Caccamo 2007 | 135 | plasma | HPLC | Total | 49 | 16.3 | 5.7 | 86 | 11.7 | 2.9 |  |
| 13 | Camicioli 2009 | 101 | plasma | CLIA | Total | 51 | 13.6 | 3.8 | 50 | 10.5 | 2.5 |  |
| 14 | Gorgone 2012 | 142 | plasma | HPLC | Total | 60 | 16.3 | 3.2 | 82 | 11.2 | 2.8 |  |
| 15 | Lee 2010 | 380 | serum | CLIA | Total | 95 | 13.0 (7.7–99.2) | | 285 | 11.5 (7.2–19.3) | |  |
| 16 | Religa 2006 | 214 | plasma | FPIA | L-Dopa-treated | 99 | 17.25 | 5.96 | 100 | 14.43 | 4.48 |  |
|  |  |  |  |  | Not L-Dopa-treated | 15 | 16.37 | 5.53 |  |  |  |  |
| 17 | Rodriguez-Oroz 2009 | 119 | plasma | SPCEI | PD-CN | 37 | 14.9 | 4.7 | 30 | 8.55 | 1.95 |  |
|  |  |  |  |  | PD-MCI | 22 | 15.1 | 4.3 |  |  |  |  |
|  |  |  |  |  | PD-D | 30 | 15.4 | 5.4 |  |  |  |  |
| 18 | Sapkota 2014 | 95 | plasma | NA | Total | 46 | 13.92 | 3.81 | 49 | 10.45 | 2.57 |  |
| 19 | Shin 2009 | 74 | NA | NA | Total | 33 | 13.6 | 7.3 | 41 | 11 | 2.9 |  |
|  |  |  |  |  | Hcy level ＞ 14 μmol/L | 12 | 20 | 8.6 |  |  |  |  |
|  |  |  |  |  | Hcy level ＜ 14 μmol/L | 21 | 9.9 | 2.2 |  |  |  |  |
| 20 | Slawek 2013 | 376 | plasma | FPIA | total | 192 | 17.76 | 7.86 | 184 | 13.6 | 7.39 |  |
|  |  |  |  |  | nPDD | 135 | 16.45 | 6.33 |  |  |  |  |
|  |  |  |  |  | nPDD-Females | 73 | 14.85 | 5.11 |  |  |  |  |
|  |  |  |  |  | nPDD-Males | 62 | 18.34 | 7.11 |  |  |  |  |
|  |  |  |  |  | PDD | 57 | 20.86 | 10.04 |  |  |  |  |
|  |  |  |  |  | PDD-Females | 28 | 20.49 | 10.71 |  |  |  |  |
|  |  |  |  |  | PD-Males | 29 | 21.22 | 9.52 |  |  |  |  |
| 21 | Song 2013 | 109 | plasma | NA | nPDD | 33 | 11.59 | 3.6 | 48 | 12.52 | 4.19 |  |
|  |  |  |  |  | PDD | 28 | 18.8 | 15 |  |  |  |  |
| 22 | Todorović 2006 | 166 | NA | NA | not L-Dopa treated | 30 | 16.93 | 7.08 | 53 | 13.13 | 4.25 |  |
|  |  |  |  |  | L-Dopa treated | 83 | 17.05 | 6.25 |  |  |  |  |
| 23 | Yuan 2009 | 186 | plasma | FPIA | L-Dopa treated | 48 | 13.16 | 4.74 | 110 | 9.8 | 2.6 |  |
|  |  |  |  |  | ≤60 | 9 | 11.04 | 4.73 | 15 | 10.1 | 2.74 |  |
|  |  |  |  |  | 61-70 | 10 | 11.32 | 2.72 | 43 | 9.24 | 2.22 |  |
|  |  |  |  |  | 71-80 | 19 | 13.06 | 4.47 | 42 | 9.9 | 2.97 |  |
|  |  |  |  |  | ＞80 | 10 | 16.6 | 5.52 | 10 | 11.3 | 1.7 |  |
|  |  |  |  |  | female | 31 | 12.29 | 4.02 | 73 | 9.14 | 2.26 |  |
|  |  |  |  |  | male | 17 | 14.45 | 5.79 | 37 | 11.1 | 2.76 |  |
|  |  |  |  |  | not L-Dopa treated | 28 | 10.08 | 2.16 |  |  |  |  |
|  |  |  |  |  | ≤60 | 5 | 8.53 | 1.03 |  |  |  |  |
|  |  |  |  |  | 61-70 | 7 | 9.11 | 1.95 |  |  |  |  |
|  |  |  |  |  | 71-80 | 14 | 11.03 | 2.19 |  |  |  |  |
|  |  |  |  |  | ＞80 | 2 | 13.06 | 0.25 |  |  |  |  |
|  |  |  |  |  | female | 17 | 9.97 | 2.4 |  |  |  |  |
|  |  |  |  |  | male | 11 | 10.69 | 2.06 |  |  |  |  |
| 24 | Wei 2016 | 102 | serum | enzyme cycling method | Total | 17 | 26.65 | 10.6 | 85 | 22.24 | 12.82 |  |
| 25 | Zou 2018 | 172 | serum | SPCEI | PDD | 92 | 16.18 | 4.96 | 80 | 10.45 | 3.19 |  |
| NA: not available; SPCEI: solid-phase chemiluminescent enzyme immunoassay; HPLC: high-performance liquid chromatography; FPIA: fluorescence polarization immunoassay technology; CLIA: direct competitive chemiluminescence immunoassays; CN: cognitively normal; MCI: mild cognitive impairment; PDD: PD patients with dementia. | | | | | | | | | | | | |

**Table S4.** Characteristics of vitamin B12 (ng/mL) in the included studies.

| **ID** | **Study** | **Sample size** | **Source of the sample** | **Method** | **Subgroups** | **Experimental groups** | | | **Control group** | | |
| --- | --- | --- | --- | --- | --- | --- | --- | --- | --- | --- | --- |
|  |  |  |  |  |  | **n** | **MEAN** | **SD** | **n** | **MEAN** | **SD** |
| 3 | Ozer 2006 | 67 | NA | CMIA | Total | 39 | 225.40 | 122.90 | 28 | 308.40 | 98.60 |
|  |  |  |  |  | Hcy level ＞ 14 μmol/L | 17 | 197 | 119.8 | 20 | 322.3 | 140.5 |
|  |  |  |  |  | Hcy level ＜ 14 μmol/L | 17 | 243.1 | 124.4 | 20 | 322.3 | 140.5 |
| 4 | Triantafyllou 2008 | 204 | serum | MPs-CILA | Total | 111 | 292.8 | 122.6 | 93 | 384.3 | 155.1 |
|  |  |  |  |  | Male | 65 | 287.9 | 135.2 |  |  |  |
|  |  |  |  |  | Female | 46 | 299.8 | 103.3 |  |  |  |
|  |  |  |  |  | Cognitive impairment | 51 | 275 | 122.2 |  |  |  |
|  |  |  |  |  | No Cognitive impairment | 60 | 308.1 | 155 |  |  |  |
| 5 | Zoccolella 2009 | 275 | serum | immunoassay | Total (L-Dopa treated) | 121 | 472 | 331 | 154 | 514 | 202 |
|  |  |  |  |  | PDD | 42 | 406 | 204 |  |  |  |
|  |  |  |  |  | nPDD | 79 | 508 | 381 |  |  |  |
| 6 | Zoccolella 2005 | 70 | serum | immunoassay | Dopamine-agonists treated | 15 | 393 | 157 | 15 | 407 | 107 |
|  |  |  |  |  | L-Dopa treated | 15 | 438 | 183 |  |  |  |
|  |  |  |  |  | L-Dopa and COMT-I treated | 15 | 439 | 248 |  |  |  |
| 7 | Lamberti2004 | 78 | serum | immunoassay | L-Dopa treated | 26 | 444 | 179 | 32 | 491 | 178 |
|  |  |  |  |  | L-Dopa and COMT-I treated | 20 | 376 | 231 |  |  |  |
| 8 | Triantafyllou 2007 | 134 | serum | MPs-CILA | Total | 67 | 295.2 | 125.8 | 67 | 380.8 | 137.4 |
| 10 | Białecka 2012 | 574 | NA | NA | Total | 320 | 332 | 142 | 254 | 400 | 204 |
|  |  |  |  |  | nPDD | 153 | 347 | 147 |  |  |  |
|  |  |  |  |  | PDD | 64 | 304 | 141 |  |  |  |
| 12 | Caccamo 2007 | 135 | plasma | HPLC | Total | 49 | 449.1 | 318.5 | 86 | 478.3 | 202.3 |
| 13 | Camicioli 2009 | 101 | plasma | CLIA | Total | 51 | 299 | 120 | 50 | 379 | 188 |
| 14 | Gorgone 2012 | 142 | plasma | Simul-TRAC-SNB 125I Radio-Immuno- Assay (RIA KIT) | Total | 60 | 418.5 | 219.8 | 82 | 440.7 | 218.5 |
| 16 | Religa 2006 | 214 | plasma | immunoassay | L-Dopa treated | 99 | 326.7 | 174.9 | 100 | 413.5 | 241.3 |
|  |  |  |  |  | not L-Dopa treated | 15 | 273 | 86.5 |  |  |  |
| 17 | Rodriguez-Oroz 2009 | 119 | plasma | SPCEI | PD-CN | 37 | 400.1 | 222.2 | 30 |  |  |
|  |  |  |  |  | PD-MCI | 22 | 353.2 | 205.6 |  |  |  |
|  |  |  |  |  | PDD | 30 | 380.2 | 156.5 |  |  |  |
| 18 | Sapkota 2014 | 95 | NA | NA | Total | 46 | 297.35 | 123.29 | 49 | 382.82 | 188.47 |
| 19 | Shin 2009 | 74 | NA | NA | Total | 33 | 716.6 | 406.2 | 41 | 883.6 | 320.2 |
|  |  |  |  |  | Hcy level ＞ 14 μmol/L | 12 | 481.3 | 124.1 |  |  |  |
|  |  |  |  |  | Hcy level ＜ 14 μmol/L | 21 | 851 | 450.9 |  |  |  |
| 21 | Song 2013 | 109 | plasma | NA | nPDD | 33 | 641.92 | 298.9 | 48 | 637.85 | 324.54 |
|  |  |  |  |  | PDD | 28 | 581.24 | 359.93 |  |  |  |
| 23 | Yuan 2009 | 186 | plasma | CLEIA | L-Dopa treated | 48 | 480.83 | 259.31 | 110 | 491.27 | 184.09 |
|  |  |  |  |  | not L-Dopa treated | 28 | 406.54 | 152.07 |  |  |  |
| 24 | Wei 2016 | 102 | serum | Radioassay kit | Total | 17 | 142.45 | 83.08 | 85 | 145.12 | 80.05 |
| NA: not available; CMIA: chemiluminescence microparticle immunoassay; SPCEI: solid-phase chemiluminescent enzyme immunoassay; HPLC: high-performance liquid chromatography; CLIA: direct competitive chemiluminescence immunoassays; MPs-CILA: paramagnetic particles-based chemiluminescence immunoassay; CLEIA: chemiluminescence enzyme immunoassay; CN: cognitively normal; MCI: mild cognitive impairment; PDD: PD patients with dementia. | | | | | | | | | | | |

**Table S5.** Characteristics of folate (ng/mL) in the included studies.

| **ID** | **Study** | **Sample size** | **Source of the sample** | **Method** | **Subgroups** | **Experimental groups** | | | **Control group** | | |
| --- | --- | --- | --- | --- | --- | --- | --- | --- | --- | --- | --- |
|  |  |  |  |  |  | **n** | **MEAN** | **SD** | **n** | **MEAN** | **SD** |
| 3 | Ozer 2006 | 67 | NA | MEIA | Total | 39 | 7.20 | 3.30 | 28 | 8.90 | 3.40 |
|  |  |  |  |  | Hcy level ＞ 14 μmol/L | 17 | 6 | 2.9 | 20 | 8.3 | 1.7 |
|  |  |  |  |  | Hcy level ＜ 14 μmol/L | 17 | 88 | 3.4 | 20 | 8.3 | 1.7 |
| 4 | Triantafyllou 2008 | 204 | serum | MPs-CILA | Total | 111 | 4.38 | 2.28 | 93 | 5.45 | 2.9 |
| 5 | Zoccolella 2009 | 275 | serum | immunoassay | Total (L-Dopa treated) | 121 | 8.3 | 5.8 | 154 | 7.5 | 11.6 |
|  |  |  |  |  | PDD | 42 | 7.9 | 5.4 |  |  |  |
|  |  |  |  |  | nPDD | 79 | 8.4 | 6.1 |  |  |  |
| 6 | Zoccolella 2005 | 70 | serum | immunoassay | Dopamine-agonists treated | 15 | 5.6 | 3.2 | 15 | 6.5 | 3.4 |
|  |  |  |  |  | L-Dopa treated | 15 | 4.8 | 21 |  |  |  |
|  |  |  |  |  | L-Dopa and COMT-I treated | 15 | 9.6 | 3.4 |  |  |  |
| 7 | Lamberti 2004 | 78 | serum | immunoassay | L-Dopa treated | 26 | 5.8 | 2.3 | 32 | 7.5 | 3.9 |
|  |  |  |  |  | L-Dopa and COMT-I treated | 20 | 8.8 | 4.8 |  |  |  |
| 8 | Triantafyllou 2007 | 134 | serum | MPs-CILA | Total | 67 | 5.02 | 2.9 | 67 | 6.98 | 3.53 |
| 10 | Białecka 2012 | 574 | NA | NA | Total | 320 | 8.9 | 4.2 | 254 | 9.5 | 4.1 |
|  |  |  |  |  | nPDD | 153 | 9.3 | 4.2 |  |  |  |
|  |  |  |  |  | PDD | 64 | 8.5 | 4.4 |  |  |  |
| 12 | Caccamo 2007 | 135 | plasma | HPLC | Total | 49 | 4.6 | 1.8 | 86 | 4.7 | 1.4 |
| 13 | Camicioli 2009 | 101 | plasma | CLIA | Total | 51 | 823 | 207 | 50 | 889 | 228 |
| 14 | Gorgone 2012 | 142 | plasma | Simul-TRAC-SNB 125I Radio-Immuno- Assay (RIA KIT) | Total | 60 | 4.5 | 1.1 | 82 | 4.9 | 1.2 |
| 16 | Religa 2006 | 214 | serum | Abbott Lab oratories AxSYM Folate Reagent assay | L-Dopa treated | 99 | 9.21 | 4.23 | 100 | 7.56 | 5.39 |
|  |  |  |  |  | not L-Dopa treated | 15 | 7.45 | 2.82 |  |  |  |
| 17 | Rodriguez-Oroz 2009 | 119 | plasma | SPCEI | PD-CN | 37 | 10.2 | 5.6 | 30 |  |  |
|  |  |  |  |  | PD-MCI | 22 | 11.6 | 6.5 |  |  |  |
|  |  |  |  |  | PDD | 30 | 10.4 | 4.6 |  |  |  |
| 18 | Sapkota 2014 | 95 | NA | NA | Total | 46 | 833.24 | 209.92 | 49 | 833.24 | 209.92 |
| 19 | Shin 2009 | 74 | NA | NA | Total | 33 | 8.5 | 4 | 41 | 10.2 | 4.8 |
|  |  |  |  |  | Hcy level ＞ 14 μmol/L | 12 | 5.6 | 1.4 |  |  |  |
|  |  |  |  |  | Hcy level ＜ 14 μmol/L | 21 | 10.1 | 4.1 |  |  |  |
| 21 | Song 2013 | 109 | NA | NA | nPDD | 33 | 15.21 | 11.34 | 48 | 17.01 | 14.82 |
|  |  |  |  |  | PDD | 28 | 13.01 | 12.01 |  |  |  |
| 23 | Yuan 2009 | 186 | plasma | CLIA | L-Dopa treated | 48 | 8.07 | 4.77 | 110 | 8.99 | 4.56 |
|  |  |  |  |  | not L-Dopa treated | 28 | 9.21 | 3.58 |  |  |  |
| 24 | Wei 2016 | 102 | serum | Radioassay kit | Total | 17 | 7.63 | 5.09 | 85 | 8.85 | 5.99 |
| NA: not available; MEIA: microparticle enzyme immunoassay; SPCEI: solid-phase chemiluminescent enzyme immunoassay; HPLC: high-performance liquid chromatography; CLIA: direct competitive chemiluminescence immunoassays; MPs-CILA: paramagnetic particles-based chemiluminescence immunoassay; CLEIA: chemiluminescence enzyme immunoassay; CN: cognitively normal; MCI: mild cognitive impairment; PDD: PD patients with dementia. | | | | | | | | | | | |

**Reference**

1. Li J, Gu C, Zhu M, Li D, Chen L, Zhu X. Correlations between blood lipid, serum cystatin C, and homocysteine levels in patients with Parkinson's disease. *Psychogeriatrics* 2020; **20**(2): 180-8.

2. Chen D, Wei X, Zou J, et al. Contra-Directional Expression of Serum Homocysteine and Uric Acid as Important Biomarkers of Multiple System Atrophy Severity: A Cross-Sectional Study. *Front Cell Neurosci* 2015; **9**: 247.

3. Ozer F, Meral H, Hanoglu L, et al. Plasma homocysteine levels in patients treated with levodopa: motor and cognitive associations. *Neurol Res* 2006; **28**(8): 853-8.

4. Triantafyllou NI, Nikolaou C, Boufidou F, et al. Folate and vitamin B12 levels in levodopa-treated Parkinson's disease patients: their relationship to clinical manifestations, mood and cognition. *Parkinsonism Relat Disord* 2008; **14**(4): 321-5.

5. Zoccolella S, dell'Aquila C, Abruzzese G, et al. Hyperhomocysteinemia in levodopa-treated patients with Parkinson's disease dementia. *Mov Disord* 2009; **24**(7): 1028-33.

6. Zoccolella S, Lamberti P, Armenise E, et al. Plasma homocysteine levels in Parkinson's disease: role of antiparkinsonian medications. *Parkinsonism Relat Disord* 2005; **11**(2): 131-3.

7. Lamberti P, Zoccolella S, Iliceto G, et al. Effects of levodopa and COMT inhibitors on plasma homocysteine in Parkinson's disease patients. *Mov Disord* 2005; **20**(1): 69-72.

8. Triantafyllou NI, Kararizou E, Angelopoulos E, et al. The influence of levodopa and the COMT inhibitor on serum vitamin B12 and folate levels in Parkinson's disease patients. *Eur Neurol* 2007; **58**(2): 96-9.

9. Saadat P, Ahmadi Ahangar A, Samaei SE, et al. Serum Homocysteine Level in Parkinson's Disease and Its Association with Duration, Cardinal Manifestation, and Severity of Disease. *Parkinsons Dis* 2018; **2018**: 5813084.

10. Białecka M, Kurzawski M, Roszmann A, et al. Association of COMT, MTHFR, and SLC19A1(RFC-1) polymorphisms with homocysteine blood levels and cognitive impairment in Parkinson's disease. *Pharmacogenet Genomics* 2012; **22**(10): 716-24.

11. Ojo OO, Oladipo OO, Ojini FI, Sanya EO, Danesi MA, Okubadejo NU. Plasma homocysteine level and its relationship to clinical profile in Parkinson's disease patients at the Lagos University Teaching Hospital. *West Afr J Med* 2011; **30**(5): 319-24.

12. Caccamo D, Gorgone G, Currò M, et al. Effect of MTHFR polymorphisms on hyperhomocysteinemia in levodopa-treated Parkinsonian patients. *Neuromolecular Med* 2007; **9**(3): 249-54.

13. Camicioli RM, Bouchard TP, Somerville MJ. Homocysteine is not associated with global motor or cognitive measures in nondemented older Parkinson's disease patients. *Mov Disord* 2009; **24**(2): 176-82.

14. Gorgone G, Currò M, Ferlazzo N, et al. Coenzyme Q10, hyperhomocysteinemia and MTHFR C677T polymorphism in levodopa-treated Parkinson's disease patients. *Neuromolecular Med* 2012; **14**(1): 84-90.

15. Lee SH, Kim MJ, Kim BJ, et al. Hyperhomocysteinemia due to levodopa treatment as a risk factor for osteoporosis in patients with Parkinson's disease. *Calcif Tissue Int* 2010; **86**(2): 132-41.

16. Religa D, Czyzewski K, Styczynska M, et al. Hyperhomocysteinemia and methylenetetrahydrofolate reductase polymorphism in patients with Parkinson's disease. *Neurosci Lett* 2006; **404**(1-2): 56-60.

17. Rodriguez-Oroz MC, Lage PM, Sanchez-Mut J, et al. Homocysteine and cognitive impairment in Parkinson's disease: a biochemical, neuroimaging, and genetic study. *Mov Disord* 2009; **24**(10): 1437-44.

18. Sapkota S, Gee M, Sabino J, Emery D, Camicioli R. Association of homocysteine with ventricular dilatation and brain atrophy in Parkinson's disease. *Mov Disord* 2014; **29**(3): 368-74.

19. Shin HW, Sohn YH. Hyperhomocysteinemia in patients with Parkinson's disease and relationship to vitamin B level. *J Mov Disord* 2009; **2**(1): 33-6.

20. Sławek J, Roszmann A, Robowski P, et al. The impact of MRI white matter hyperintensities on dementia in Parkinson's disease in relation to the homocysteine level and other vascular risk factors. *Neurodegener Dis* 2013; **12**(1): 1-12.

21. Song IU, Kim JS, Park IS, et al. Clinical significance of homocysteine (hcy) on dementia in Parkinson's disease (PD). *Arch Gerontol Geriatr* 2013; **57**(3): 288-91.

22. Todorović Z, Dzoljić E, Novaković I, et al. Homocysteine serum levels and MTHFR C677T genotype in patients with Parkinson's disease, with and without levodopa therapy. *J Neurol Sci* 2006; **248**(1-2): 56-61.

23. Yuan RY, Sheu JJ, Yu JM, et al. Methylenetetrahydrofolate reductase polymorphisms and plasma homocysteine in levodopa-treated and non-treated Parkinson's disease patients. *J Neurol Sci* 2009; **287**(1-2): 64-8.

24. Wei Z, Tiandong W, Yang L, et al. Parkinson's Disease and Homocysteine: A Community-Based Study in a Folate and Vitamin B12 Deficient Population. *Parkinsons Dis* 2016; **2016**: 9539836.

25. Zou J, Chen Z, Liang C, et al. Trefoil Factor 3, Cholinesterase and Homocysteine: Potential Predictors for Parkinson's Disease Dementia and Vascular Parkinsonism Dementia in Advanced Stage. *Aging Dis* 2018; **9**(1): 51-65.
